# Current Global Trends in Prepectoral Breast Reconstruction

**DOI:** 10.3390/medicina60030431

**Published:** 2024-03-03

**Authors:** Saima Taj, Ravi Chandavarkar, Raghavan Vidya

**Affiliations:** The Royal Wolverhampton N.H.S. Trust, Wolverhampton Road, Wolverhampton WV10 0QP, UK; s.taj1@nhs.net (S.T.); ravi.chandavarkar1@nhs.net (R.C.)

**Keywords:** prepectoral breast reconstruction, implant-based breast reconstruction, mastectomy, complications, acellular dermal matrix, synthetic mesh, radiotherapy, fat grafting

## Abstract

Implant-based breast reconstruction (IBBR) is the most frequently performed procedure for breast reconstruction following mastectomy, which involves the surgical placement of breast implants. The approach to breast reconstruction can be divided into two main categories, namely prepectoral breast reconstruction (PPBR) and subpectoral breast reconstruction (SPBR), based on the implant plan and placement technique. In recent years, there has been a significant surge in the popularity of prepectoral implant-based breast reconstruction, where the implants are positioned above the chest muscle, as opposed to beneath it in the subpectoral approach. However, despite this growing preference, there is a lack of comprehensive data regarding the national trends in the utilization of this technique, thus necessitating further investigation. This narrative review aims to ascertain the current global patterns linked to prepectoral breast reconstruction and elucidate the considerations surrounding patient and implant selection, reconstructive techniques, the utilization of meshes in prepectoral reconstruction, the ensuing outcomes and complications, the ramifications of radiotherapy, and the potential advantages of integrating fat infiltration into the implementation of this technique in breast reconstruction with a focus on published papers in last five years. Conclusion: Prepectoral breast reconstruction has emerged as an appropriate surgical option for individuals seeking breast reconstruction. This development can be attributed to the recent progress made in implant technology, which has significantly enhanced the outcomes of this procedure. Additionally, advancements in mastectomy techniques, autologous fat grafting, and the use of acellular dermal matrices (ADMs) have also played a vital role in improving the aesthetic results of prepectoral breast reconstruction. As a result, the significance and effectiveness of this technique in the field of breast reconstruction have been firmly established, making it an essential component of the overall armamentarium available to plastic surgeons for breast reconstruction purposes.

## 1. Introduction

In the United Kingdom, fifty-five thousand women are diagnosed with breast cancer on an annual basis, and it has been found that 40% of these women require a mastectomy [1]. The removal of a breast can have a profound impact on the overall quality of life for these women, leading to psychological inferiority and a decrease in social integration [1,2]. As part of the comprehensive treatment for breast cancer, breast reconstruction plays a pivotal role as it not only enhances the aesthetic appearance but also restores the natural contours of the breast [3]. The predominant approach utilized in the United Kingdom for immediate reconstructions after mastectomy is an implant-based reconstruction (IBBR), accounting for nearly 70% of all cases [4]. When it comes to the placement of the implant, there are two categories to consider: prepectoral breast reconstruction (PPBR) and subpectoral breast reconstruction (SPBR) [2]. Prepectoral breast reconstruction was initially introduced in the early 1960s and showed great promise in its early results [5]. However, it was accompanied by high complication rates, such as skin flap necrosis, significant instances of capsular contracture, implant extrusion, poor aesthetic outcomes, and infection [6]. The findings from various research studies have led surgeons to shift their focus towards placing the implants beneath the pectoral muscles, as this approach provides better coverage and effectively prevents certain complications [7,8]. Nevertheless, subpectoral implant placement often leads to persistent muscle pain, muscle spasms or contractions, animation deformity, decreased mobility in the upper extremity, and ultimately a decline in the patient’s physical strength [9,10]. In recent years, there has been an increasing preference for prepectoral implant-based breast reconstruction due to advancements in implant technology, improved mastectomy techniques, the incorporation of autologous fat grafting, and the utilization of acellular dermal matrices (ADMs). However, available data are scarce regarding the national trends in the utilization of this technique. This study aimed to determine practice patterns related to prepectoral breast reconstruction. Therefore, we conducted a narrative review on prepectoral breast reconstruction. Specifically, we focused on the crucial aspects of patient and implant selection, reconstructive techniques, prepectoral reconstruction with or without meshes, the subsequent outcomes and complications, the impact of radiotherapy, and the potential benefits of incorporating fat infiltration as a scaffolding technique. The electronic database PubMed, Scopus, and the Cochrane Library Central Register of Controlled Trials were searched for studies on post-mastectomy patients undergoing prepectoral breast reconstruction using the terms ‘breast reconstruction’, ‘mastectomy’, ‘pre pectoral’ or ‘pre-pectoral’ ‘implant-based breast reconstruction’, ‘complications’, ‘acellular dermal matrix’, ‘synthetic mesh’, ‘without meshes’, ‘radiotherapy’, and ‘fat grafting’. The search was carried out in September 2023 including papers from January 2019 to November 2023. Studies not written in English, animal studies, and case reports were excluded.

## 2. Evolution of the Prepectoral Breast Reconstruction Technique

The prepectoral method of breast reconstruction has undergone significant development and is now widely regarded as the most popular approach following mastectomy. This is due to its numerous advantages to carefully selected patients [11]. This technique involves the placement of the implant above the pectoralis major muscle, either with or without reinforcement from the mesh. By sparing the pectoralis major muscle, the prepectoral method minimizes many undesirable consequences typically associated with dual-plane breast reconstruction, such as animation, discomfort resulting from muscle spasms, and implant lateralization [6,7]. Additionally, the prepectoral approach provides the added benefit of optimal positioning of the prosthetic device on the chest wall, allowing it to closely resemble the appearance of a natural breast.

The iBRA prospective multicenter cohort study has yielded valuable insights into the short-term complications associated with both prepectoral and subpectoral reconstruction methods. Interestingly, the study found that the complications observed in both methods were comparable. Furthermore, after 18 months, patients who underwent prepectoral reconstruction may experience a higher level of satisfaction with the outcome, as determined by the validated BREAST-Q assessment tool, in comparison to those who underwent subpectoral techniques [12].

Prepectoral breast reconstruction, a technique known for its safety and efficacy, has been widely recognized for its ability to deliver satisfactory oncological and aesthetic results [11,13,14]. However, due to the placement of the breast implant directly beneath the skin, which is known to have a relatively lower level of vascularity, there is a potential for complications to arise. As a result, it becomes imperative to exercise caution when selecting patients for this technique, ensuring that they are suitable candidates. Additionally, it is crucial to thoroughly evaluate the intraoperative mastectomy flap to guarantee optimal outcomes. To achieve this, it is recommended to follow a well-established pathway specifically designed for prepectoral reconstructions, which can provide guidance and minimize the risk of potential complications [11].

## 3. Patient Selection

Patient selection holds a position of utmost importance, as it has been widely recognized that the presence of risk factors is strongly correlated with unfavorable outcomes. This recognition highlights the critical nature of carefully choosing individuals who are most suitable for the procedure. In PPBR, one vital aspect of this approach involves ensuring the availability of a robust and sufficiently vascularized mastectomy flap. This requirement ensures that the reconstructed breast has an adequate blood supply, which is essential for its long-term success.

Furthermore, the proximity of the implant to the skin surface introduces an additional factor to consider in patient selection. This proximity poses a potential risk for skin-related complications that could compromise the overall success of the reconstruction. Therefore, it is imperative to carefully evaluate the suitability of individuals for this procedure based on their physical condition, medical history, and specific risk factors.

The procedure is exclusively offered to individuals who meet certain criteria. Firstly, these individuals must be in good physical condition, without any significant comorbidities or well-managed comorbidities. This ensures that they have the necessary physical resilience to undergo the procedure and recover successfully. Additionally, individuals with a body mass index (BMI) below 35 are preferred candidates for this procedure. This criterion is important because higher BMIs have been associated with an increased risk of complications during and after surgery.

Moreover, individuals with no history of previous radiotherapy damage are considered more suitable candidates for PPBR. The presence of previous radiation damage can complicate the surgical process and increase the risk of complications. Similarly, individuals with a resectable tumor are preferred candidates for this procedure.

Nevertheless, certain conditions may be considered relative contraindications for PPBR. For example, individuals with an elevated BMI (below 40), uncontrolled diabetes mellitus, active smoking, immunosuppression, and previous radiation damage are at an increased risk of perioperative complications. As a result, these individuals should be carefully evaluated to determine the feasibility and potential risks of the procedure.

In addition to these relative contraindications, there are specific cases where PPBR should be avoided altogether. These cases include situations where the tumors involve the skin, chest wall muscle, locally advanced tumors, inflammatory breast cancers, or tumors with a heightened likelihood of chest wall recurrence. In these instances, alternative treatment options should be considered to ensure the best possible outcomes for the patient [15,16,17].

## 4. Reconstructive Technique

PPBR can be conducted as either a single-stage or two-stage procedure for breast tissue expansion [15]. However, the choice of technique primarily depends on factors such as the quality of the mastectomy flaps, the presence of risk factors, the necessity for adjuvant therapy, and the patient’s preference for postoperative breast size [5,15,16,18]. Two-stage reconstruction is generally considered safer when risk factors are identified [15], as it allows for gradual expansion of the skin envelope. However, it does require additional surgeries and a longer completion time for the reconstruction of a patient [19].

Moreover, the advantages of two-stage reconstruction include better control over the final position, size, and shape of the implant, as well as a reduced risk of wound healing complications. Additionally, it provides the opportunity for routine fat grafting during the second stage [15,20]. Nevertheless, there are disadvantages associated with two-stage reconstruction, such as the discomfort of filling the expander, potential injury to the expander, and a longer time until final results are achieved. On the other hand, single-stage reconstruction has its own disadvantages, including the inability to make fine adjustments to implant size and positioning, as well as increased stress on potentially compromised mastectomy skin flaps [20]. It is worth noting that one-stage reconstruction is the standard approach in Europe, although there has been an increase in the incidence of one-stage IBBR in the United States in recent years (2016: 10.8% vs. 2019: 17.8%). However, most breast reconstruction procedures in the United States remain two-stage (2016: 89.2% vs. 2019: 82.2%), according to recent statistics from the American Society of Plastic Surgeons [21].

The decision regarding whether to opt for conventional skin-sparing or nipple-sparing mastectomy incisions can be determined by considering the desired shape of the breast as well as the necessity for reducing the amount of skin. It is generally advisable to strategize incisions in a manner that minimizes any disturbance to the subcutaneous vasculature. Incisions made around the areola are thought to carry a greater degree of risk, and it is crucial to plan all incisions in such a way that allows for a closure involving two layers and double-breasting. This approach ensures optimal outcomes and reduces the likelihood of complications [22].

## 5. Implant Selection in Prepectoral Breast Reconstruction

The primary objective of breast reconstruction is to replicate a breast that possesses the appearance and tactile qualities of a natural breast [23]. However, the development of silicone gel technology in fourth-generation and cohesive gel in fifth-generation breast implants has led to the production of implants that are both safer and exhibit a more authentic appearance [5]. All contemporary silicone implants are cohesive in nature, meaning that the silicone filler is not in the form of liquid silicone but rather in the form of viscous silicone. These highly cohesive implants can maintain their shape and dimensions, indicating that the distribution of gel within the implant remains unchanged when the implant is held in either a vertical or horizontal position. Anatomically shaped implants, specifically, are considered to be form-stable implants with high cohesiveness. These implants possess a textured surface, which serves to decrease the likelihood of implant rotation. Due to their higher viscosity, form-stable implants are more rigid in comparison to less cohesive implants, resulting in a reduced risk of rippling. Conversely, round-shaped implants are available in both textured and smooth surfaces and exhibit varying degrees of gel cohesivity, ranging from cohesive to highly cohesive. Implants with lower cohesivity are softer than those with higher cohesivity, yet they are associated with an increased risk of rippling.

The process of selecting the appropriate implant can prove to be overwhelming for both patients and surgeons alike. Nevertheless, the selection of implants should be guided by fundamental principles that revolve around tissue-based measurements, the extent of soft tissue thickness (determined through the soft tissue pinch test in the medial upper pole of the breast), and the desires of the patient. The thickness of soft tissue coverage is utilized to inform the decision between less or more cohesive implants. In cases where patients possess thick, soft tissue coverage, a less cohesive implant is typically a more suitable choice. Conversely, in patients with thin, soft tissue coverage, a more cohesive implant is deemed to be a better fit, irrespective of the shape of the implant (whether it be round or anatomical) [23].

## 6. Prepectoral Breast Reconstruction and Use of Indocyanine Green Angiography

Regardless of the technique employed for implant-based breast reconstruction, the most dreaded complication that surgeons encounter is mastectomy skin flap necrosis [5,24]. Therefore, the preservation of this subcutaneous layer and its vascular supply is crucial for the success of the surgery. To some extent, the thickness of the subcutaneous layer can be assessed prior to the operation using digital mammography or magnetic resonance imaging [25,26]. However, the true test of the viability of the skin flap occurs during the surgery itself. Traditionally, surgeons have assessed the vascularity of the mastectomy skin flap based on its color, absence of dermal exposure, and damage caused by diathermy [15].

In recent years, a new tool called indocyanine green angiography (ICGA) has emerged as a useful adjunctive technique to evaluate tissue perfusion and thereby decrease the incidence of mastectomy flap necrosis [27,28]. A meta-analysis published in the Cochrane database by Pruimboom et al. demonstrated that the use of ICGA can significantly reduce the need for flap repair after breast reconstruction [29]. However, it is important to note that there is still a lack of high-quality evidence supporting the routine use of ICGA in assessing mastectomy skin flap necrosis. Therefore, further high-quality randomized controlled studies comparing the use of ICGA to clinical evaluation are needed to establish its effectiveness and superiority [3].

## 7. Prepectoral Breast Reconstruction with Biological Meshes

The use of ADMs and synthetic meshes in prepectoral breast reconstruction has revolutionized the field, offering improved outcomes and patient satisfaction. The protective layer created by these adjuncts not only safeguards the implant but also contributes to the overall aesthetic result of the procedure. Surgeons must carefully consider the selection and placement of these adjuncts to ensure optimal results.

One of the key aspects of these adjuncts is their ability to create a protective layer of tissue between the mastectomy flap and implant. This layer serves multiple purposes, including shielding the implant from exposure and ensuring its stability. Additionally, it effectively prevents any lateral migration of the implant, which is of utmost importance for successful reconstruction [30,31].

The alloplastic adjuncts available in the market are diverse, providing a wide range of options for surgeons. These adjuncts can be derived from various sources, including human, animal, and synthetic devices. This variety allows surgeons to select the most suitable ADM based on the patient’s specific needs and preferences. Furthermore, these ADMs can be further categorized based on their meshed or fenestrated structure, each having its unique characteristics and properties [7,32].

When it comes to integration, ADM meshes are effectively incorporated through a process of remodeling and neovascularization. On the other hand, synthetic meshes achieve integration through fibroblastic and foreign body reaction mechanisms [33]. However, it is important to acknowledge that these adjuncts are not without their complications. Infection, seroma formation, and red breast syndrome are potential risks associated with their use [34]. Nevertheless, ADMs and meshes have proven to be valuable assets in preventing implant-associated complications in prepectoral immediate breast reconstruction (IBBR) [35]. Complications such as upper pole rippling, capsular contracture, and the risk of mastectomy skin flap failure can be significantly reduced with their utilization [30,36].

Despite the benefits of ADMs, it is crucial to recognize that their use does not eliminate the possibility of complications in prepectoral breast reconstruction. Therefore, it is imperative to exercise meticulous patient selection and carefully consider the different varieties of ADMs available. In a study conducted by Tellarini et al., it was found that individuals undergoing ADM-assisted prepectoral breast reconstruction are more prone to complications if they smoke, undergo adjuvant radiotherapy or axillary lymph node dissection, or have a larger breast volume. The relationship between diabetes, high BMI, and breast implant size as potential risk factors is still a topic of debate [32].

To further evaluate the impact of ADMs, a meta-analysis of 23 studies conducted by Lee and Mun demonstrated that the use of ADMs significantly decreases the occurrence of capsular contracture and implant malposition [37]. This finding highlights the positive effect these adjuncts can have in improving outcomes and reducing complications in prepectoral breast reconstruction. However, it is important to continue researching and exploring the potential risks and benefits associated with ADMs to optimize patient outcomes and ensure the highest level of safety and efficacy in prepectoral breast reconstruction procedures.

## 8. Prepectoral Breast Reconstruction with Non-Biological Meshes

In addition, as an alternative to the utilization of acellular dermal matrices (ADMs), the utilization of meshes has demonstrated the ability to yield aesthetically pleasing outcomes when employed in prepectoral breast reconstruction (PPBR), while their porous composition aids in the mitigation of seroma formation. Furthermore, meshes prove to be a more economical option in comparison to ADMs [38,39]. Meshes serve as a resilient medium that seamlessly integrates into the tissue expander or implant pocket, minimizing complications such as implant migration and capsular contracture, much like ADMs [7]. The recent studies on prepectoral breast reconstruction with biological and non- biological meshes presented in Table 1.

## 9. Prepectoral Breast Reconstruction without Meshes

There exists a wide range of technical variations in the field of prepectoral prosthesis placement. Nonetheless, most of the studies found in the literature employ acellular dermal matrices (ADMs) in some capacity. These studies as mentioned earlier have attributed numerous advantages to the utilization of ADMs, including but not limited to providing support to the prosthetic device and defining the pocket, reducing rippling, minimizing the inflammatory response, potentially lowering the rates of capsular contracture, and safeguarding against the detrimental effects of radiation. Nevertheless, some studies advocate for the prepectoral placement of a definite implant without the use of ADMs. Despite the literature mentioned above, comparative studies are scarce [43]. Currently, there is a heightened focus on the preservation of the mastectomy skin flap, the evaluation methods of the perfusion of the mastectomy flap, as well as changes in implant characteristics.

Furthermore, recently, micro polyurethane-foam-coated implants have been suggested as a viable option for prepectoral breast reconstruction (PPBR) due to their proposed ability to provide mesh-like support without necessitating the use of additional ADMs. During the consideration of implant selection, it is crucial to consider the potential risk of Breast Implant-Associated Anaplastic Large Cell Lymphoma (BIA-ALCL). While the risk is relatively low, some studies have suggested that the highest risk of BIA-ALCL is associated with the use of polyurethane implants. On the other hand, others believe that no definitive conclusions can be drawn at this time and that further surveillance and research are necessary before stating an increased risk of BIA-ALCL. However, it is important to note that there is still a need for larger and higher-quality studies that compare the technique of prepectoral breast reconstruction both with and without the use of ADMs in order to draw any definite conclusions [20].The latest research on prepectoral breast reconstruction without meshes is illustrated in Table 2.

## 10. Prepectoral Breast Reconstruction and Impact of Radiotherapy

Radiation therapy is an imperative requirement for approximately 40% of patients who have undergone mastectomy, as it plays a crucial role in their treatment plan. Post-mastectomy radiation therapy (PMRT) is an indispensable component of this treatment plan, as it serves the purpose of effectively controlling locoregional recurrence and enhancing the rate of disease-free survival for patients with locally advanced breast cancer [47,48,49]. However, it is important to acknowledge that despite the therapeutic benefits of PMRT, its implementation may result in profoundly adverse effects within the context of implant-based reconstruction. This is primarily because PMRT leads to a reduction in both the quantity and quality of microvascular blood flow to the breast, which in turn has detrimental effects on the integrity of the skin flaps and the overall condition of the breast tissue [47,50,51]. Consequently, patients may experience radiation-induced damage that becomes evident within a few days to weeks after treatment, manifesting as edema, inflammation, and desquamation on the skin and tissue of the breast. These immediate effects can give rise to complications such as wound dehiscence, infection, delayed healing, seroma, and hematoma after breast reconstruction.

As time progresses, the effects of radiation become more pronounced, leading to gradual fibrosis and atrophy of the skin and underlying subcutaneous tissues. This results in thickening of the skin, discoloration, retraction induration, and decreased breast volume. These delayed consequences of radiation can further exacerbate complications such as capsular contracture, implant loss, malposition, and distortion of the breast contour following reconstructive surgery [52,53].

To address these concerns, a retrospective review conducted by Sigalove et al. aimed to investigate the feasibility and outcomes of prepectoral reconstruction in the setting of PMRT. The study found that prepectoral reconstruction was well tolerated, with a low complication rate. This included a major surgery rate of 2.9%, a reconstructive failure rate of 2.9%, and a clinically significant capsular contracture rate of 0%. Notably, reconstructions were successfully completed in 97% of irradiated breasts. While there were no complications observed in nonirradiated breasts, the difference in the rate of complications between the irradiated and nonirradiated groups was found to be statistically nonsignificant [52]. These findings suggest that prepectoral reconstruction may be a viable option for patients undergoing PMRT, as it demonstrates promising outcomes with minimal complications.

Moreover, the timing of PMRT, specifically whether the expander or implant is irradiated, appears to have little influence on postoperative outcomes. This assertion is supported by the Mastectomy Reconstruction Outcomes Consortium Study, which reported no significant difference in complication rates between expander or implant irradiation. The study concluded that the timing of PMRT is not a significant predictor of any complication, major complication, or reconstructive failure [52,53,54,55,56,57,58]. These findings provide valuable insights into the optimal approach for PMRT in the context of reconstructive surgery, highlighting the importance of considering various factors such as the type of reconstruction and the timing of radiation therapy. By carefully evaluating these factors, healthcare professionals can make informed decisions that maximize the benefits of PMRT while minimizing the risk of complications.

However, the utilization of acellular dermal matrices (ADMs) has been under consideration even in patients who are at a heightened risk for complications, such as patients undergoing post-mastectomy radiation therapy (PMRT), who are susceptible to the development of capsular contracture [32,59,60]. Moreover, in a study conducted by Polotto et al. in the year 2023, this retrospective analysis encompassed a sample size of 485 patients, with 439 individuals undergoing ADM-assisted prepectoral breast reconstruction (PPBR). Among these patients, one group received PMRT while the other did not. The findings of this study revealed a significantly increased incidence of capsular contracture among those who underwent PMRT. Additionally, it was observed that patients who were treated with PPBR and underwent PMRT exhibited a low rate of complications as well as a mere 4.8% requirement for revisional surgery over an average follow-up duration of 35.6 months. These results suggest that the utilization of PPBR is both feasible and safe in the context of PMRT [61]. Nevertheless, the recent retrospective study revealed that patients in the radiotherapy cohort exhibited a greater incidence of capsular contracture and implant loss compared to those in the non-radiation group. Nonetheless, the frequency of complications remained within an acceptable spectrum, and by providing patients with comprehensive preoperative information, they can experience enhanced advantages from immediate reconstruction, thereby displaying an exceptional overall quality of life, regardless of radiation [62].

Furthermore, it is imperative to consider the principles of technique to attain favorable outcomes in the context of PMRT. The timing of PMRT administration, subsequent to complete tissue healing and recovery from the surgical intervention, plays a crucial role in minimizing the risk of wound dehiscence and skin necrosis. The occurrence of wound dehiscence can be mitigated through the implementation of an inframammary incision, which is deemed the preferred incision in all two-stage reconstructions. Furthermore, when planning for expander irradiation, it is recommended to complete tissue expansion prior to initiating radiation therapy [52,63]. The recent literature on the consequences of radiotherapy is exhibited in Table 3.

## 11. Scaffold with Fat Infiltration

Fat infiltration thickens the soft tissue coverage of the inserted implant, thereby enhancing the aesthetic outcome of implant-based breast reconstruction (IBBR). This leads to a reduction in complications such as implant rippling or contour irregularities, ultimately contributing to improved overall patient satisfaction post-breast cancer surgery and IBBR. It is worth noting that numerous studies have unequivocally demonstrated the significant benefits of this procedure [7].

Moreover, the oncological safety of fat grafting after breast cancer has been extensively investigated and validated through various research studies [65,66]. In a recent meta-analysis conducted by Li M and colleagues, a comprehensive review of seventeen studies encompassing a total of 7494 patients was undertaken. The objective of this metanalysis was to examine the potential disparities in outcomes between autologous fat grafting and control groups. The analysis of the observed outcomes from these studies revealed that there were no statistically significant differences in the risks of local and regional recurrence or distant metastases between autologous fat grafting and the control group [66].

The achievement of a natural-looking result in IBBR is contingent upon multiple factors, including adequate tissue coverage, appropriate implant selection, and a suitable autologous ratio. As such, the utilization of fat grafting techniques can serve to augment mastectomy skin flaps, thereby facilitating better implant coverage and enhancing the overall cosmetic outcome of the procedure [67].

It is crucial to consider that the retention of a fat graft is considerably influenced by the condition of the graft bed and the quality of the purified fat. The rates of retention can exhibit significant variability, ranging from 20% to 80%. To ensure optimal outcomes, a syringe equipped with a small-diameter cannula is utilized to inject fat tissue, typically characterized by a diameter of less than 2 mm. This specific technique, often referred to as microribbons, involves the injection of fat into separate planes, thereby promoting a higher retention rate while concurrently minimizing the occurrence of local complications, such as calcifications and oil cyst formation [68].

In the context of breast reconstruction, the prepectoral pocket has emerged as the most logical and clinically sound plan for achieving the desired breast volume. The capsule, an integral component of this approach, assumes a pivotal role as it not only defines the newly created space but also establishes a vital vascular network. By adopting a multi-session approach, it becomes feasible to successfully augment this encapsulated space through the administration of fat injections. Remarkably, the injection of a mere 100 mL of fat can yield a substantial volume augmentation of approximately 50–60 mL, underscoring the efficacy and practicality of this technique [67].

In situations where breasts have been exposed to irradiation, it is often necessary to perform fat grafting to enhance the overall appearance of the breasts. This procedure is typically carried out approximately 3–6 months after post-mastectomy radiation therapy (PMRT). However, the authors of this study are currently considering the possibility of performing fat grafting at an earlier stage. By doing so, they believe that they can take advantage of the compromised tissue perfusion caused by the irradiation process, which in turn can lead to better retention and regeneration of the grafted fat cells. Furthermore, there is some evidence suggesting that performing fat grafting earlier may help to reduce the occurrence of postoperative complications [69,70]. In a study conducted by Debald et al., it was demonstrated that fat grafting after radiotherapy resulted in a significant improvement in the quality of the skin and the regeneration of the underlying tissue [71].

The strength of this study lies in its ability to provide a review of the latest advancements in surgical techniques that have surfaced in recent years. This study specifically focuses on the unique aspects associated with this particular procedure such as the selection of patients and implants, various available prepectoral techniques, the challenges and considerations regarding radiotherapy, and the implementation of prepectoral breast reconstruction, with or without the utilization of mesh and fat grafting. However, the main limitation of this review is that it is not a systematic review and includes only recent studies; therefore, the available follow-up is often relatively short.

Currently, there exists a limited number of randomized clinical trials (RCTs) investigating prepectoral breast reconstruction, thus rendering the current body of evidence in this area somewhat insufficient. It is anticipated that in the future, a larger number of well-designed and rigorous studies will be conducted to further investigate and substantiate the safety and clinical efficacy of this surgical approach. Despite this limitation, it is noteworthy that prepectoral breast reconstruction has already demonstrated promising and favorable initial outcomes on a global scale. Nevertheless, it is imperative to acknowledge that the attainment of long-term follow-up data is vital in order to conclusively affirm and validate the sustained benefits and overall success of this particular reconstructive technique.

## 12. Conclusions

Prepectoral breast reconstruction has become a valid surgical alternative for breast reconstruction, with the recent advancement in implant technology, improved mastectomy techniques, autologous fat grafting, and acellular dermal matrices (ADMs) providing good cosmesis. Its pivotal role in breast reconstruction is now well established and forms an important armamentarium in breast reconstruction.

## Figures and Tables

**Table 1:** Prepectoral breast reconstruction with biological and non-biological meshes.

Study	Year	Findings
Pires et al. [40]	2022	This study compared bovine, porcine, and human ADMs in PPBR and showed similar complication rates except for bovine ADM, which has the highest proportion of complications
Lee et al. [37]	2023	This study illustrates that PPBR capsular contracture and implant malposition were significantly decreased with the use of ADMs
Tellarini et al. [32]	2023	This study demonstrates that complications associated with different animal-derived ADMs are generally comparable
Movassaghi et al. [41]	2023	This study showed that absorbable mesh Poly-4-hydroxybutyrate is safe and effective in PPBR and appears to have equal, if not reduced, capsular contracture rates when compared to the published data on the use of ADMs, and they are also cost-effective.
Mookerjee [42]	2023	The comparison between PPBR with vicryl mesh and PPBR with ADM showed that vicryl caused a lower rate of complications and was safer, faster, and cheaper as compared to ADM

PPBR—prepectoral breast reconstruction, ADM—acellular dermal matrix.

**Table 2:** Prepectoral breast reconstruction without meshes.

Study	Year	Findings
Salibian et al. [43]	2021	Immediate two-stage PPBR with tissue expanders has comparable rates of short-term complications with or without ADM support
Delong et al. [44]	2021	PPBR with and without mesh assistance found no difference between groups in hematoma, infection, or explanation rates, whereas capsular contracture and seroma rates were higher in the no-mesh cohort
Piers et al. [45]	2023	Found no significant differences in outcomes between prepectoral breast reconstruction with and without acellular dermal matrix
Chen et al. [46]	2023	PPBR with no biosynthetic scaffold had the lowest rates of capsular contracture and may provide the most optimal balance between economic and clinical considerations in implant-based reconstruction

**Table 3:** Impact of radiotherapy on prepectoral breast reconstruction.

Study	Year	Findings
Apte A et al. [64]	2019	The study looked at implant-based reconstruction with ADM with and without radiotherapy; this showed no significant difference in the revision surgeries in the two groups. However, the rate of capsular contracture was higher in the PMRT group
Zugasti et al. [57]	2021	This study showed significantly higher rates of surgical complications, reoperation rates, and reconstruction failure among patients who underwent PMRT after immediate IBBR. They reported a decreased level of satisfaction and unfavorable aesthetic results attributable to PMRT
Awadeen et al. [47]	2023	This study illustrates a significantly higher rate of wound infection, capsular contracture, implant loss, and poor aesthetic results among patients with PPBR and PMRT. However, there was no significant difference between PMRT and non-PMRT for wound dehiscence, seroma, hematoma, nipple necrosis, and breast rippling
Polloto et al. [61]	2023	This study demonstrates that ADM-assisted PPBR patients who underwent PMRT presented a low complication rate and minimal need for revisional surgery in the long-term follow-up, suggesting that this technique is also feasible and safe in the PMRT context

PPBR—prepectoral breast reconstruction, ADM—acellular dermal matrix, PMRT—post-mastectomy radiation therapy.
